# Assessment of Occupational Health and Safety Hazards in Mosquito Control Personnel in North Carolina and Virginia, USA

**DOI:** 10.3390/ijerph23060819

**Published:** 2026-06-19

**Authors:** Naina Sharma Bastakoti, Stephanie L. Richards, Avian White, Jo Anne Balanay

**Affiliations:** Department of Health Education and Promotion, College of Health and Human Performance, East Carolina University, Greenville, NC 27858, USA; nainas25@ecu.edu (N.S.B.); whiteav15@ecu.edu (A.W.); balanayj@ecu.edu (J.A.B.)

**Keywords:** occupational health and safety, employee health, mosquito control

## Abstract

**Highlights:**

**Public health relevance—How does this work relate to a public health issue?**

- This work informs the protection of the occupational health and safety of mosquito control personnel who encounter a variety of potential health and safety issues such as arthropod stings and bites, insecticides, heavy equipment, noise, heat, and solar ultraviolet radiation.
- The prevention of mosquito-borne diseases through targeted mosquito control is an important globally relevant public health issue.

**Public health significance—Why is this work of significance to public health?**

- This is the first known study to evaluate health and safety training and worker risk perception in mosquito control personnel in the United States.
- Workplace hazards should be evaluated to protect occupational health.

**Public health implications—What are the key implications or messages for practitioners, policy makers and/or researchers in public health?**

- Knowledge of health and safety hazards, proper risk assessment methods, and personal protective equipment will reduce and/or eliminate workplace risk.
- It is important to identify potential hazard-creating situations to tailor personal protective equipment and worker training appropriately.

**Abstract:**

Mosquito control personnel work within health departments, public works, private companies, and other agencies. These essential outdoor workers have highly specialized training and are faced with a variety of potential health and safety hazards (e.g., arthropod bites and stings, exposure to insecticides and other chemicals, working with heavy equipment, noise, heat, solar ultraviolet radiation, slips, trips, and/or falls). Mosquito control personnel undergo employer-provided and other types of training on a variety of topics from regulatory updates to new surveillance and control techniques that are required for safety purposes and to maintain their applicator license. Here, an exploratory baseline survey was conducted among members of the North Carolina Mosquito and Vector Control Association (NCMVCA) and the Virginia Mosquito Control Association (VMCA). There was a 28% response rate so results should be interpreted with caution in this pilot study. Most respondents reported utilizing ultra-low volume insecticide application equipment for controlling adult mosquitoes. Backpack sprayers were utilized by less than half of respondents. Those who reported using respirators showed higher concern about insecticide-related health effects than those who did not use respirators. Outdoor workers encounter various potential hazards and utilize several forms of personal protective equipment to reduce risks. This baseline work can be considered a starting point for implementing and strengthening occupational safety and health awareness and preventive measures for mosquito control workers. Knowledge of health and safety hazards can reduce workplace risk.

## 1. Introduction

Mosquitoes are a nuisance due to their blood feeding behavior and can be competent vectors of pathogens that cause diseases like dengue, Eastern equine encephalitis, Japanese encephalitis, malaria, Western equine encephalitis, West Nile encephalitis, and yellow fever [1]. Vector-borne diseases kill and/or debilitate millions of people globally each year [2]. Current Integrated Mosquito Management (IMM) methods carried out by mosquito control personnel include tasks such as oviposition site source reduction, mosquito and sentinel animal surveillance, as well as biological and chemical control tactics that target immature and adult mosquitoes [3,4]. Mosquito control personnel are highly trained with specialized knowledge in mosquito biology, genus and species identification, immature and adult mosquito surveillance, pathogen detection techniques, assessment of insecticide resistance, application of formulated insecticide products, equipment maintenance, community education, and monitoring federal/state/local regulations, among other important skills.

The Environmental Protection Agency (EPA) Pesticide Program conducts risk assessments and (if necessary) risk management for pesticides utilized in the United States [5]. Mosquito control personnel must be licensed to apply insecticides in their respective area with guidelines varying between states [6]. In North Carolina (NC), pesticide applicator licensing occurs through the Department of Agriculture and Consumer Services (DACS), Structural Pest Control and Pesticides Division [7]. In VA, licensing occurs through the Office of Pesticide Services [8]. Continuing education is required to maintain the applicator license, stay informed on insecticide-related regulatory changes, and keep up with new information on the surveillance and epidemiology of endemic and emerging mosquito-borne diseases, proper insecticide application techniques, and other topics.

Due to the outdoor nature of their work, mosquito control personnel are exposed to potential occupational health and safety hazards (e.g., vectors, other stinging and/or biting arthropods, snakes, noise from equipment, heat, ultraviolet (UV) radiation, insecticides, other chemicals). They also face potential ergonomic hazards from carrying heavy equipment such as backpack sprayers or handheld foggers [9]. Neuromuscular and/or musculoskeletal ailments (e.g., back pain) may result from carrying a backpack for an extended period of time; hence, weight and duration of backpack use should be considered when designing daily work plans [10,11]. Furthermore, vibrations (i.e., from combustion engines) transferred to the head, spine, and/or hands can be uncomfortable and ultimately cause fatigue, musculoskeletal, and/or physiological health problems [12,13].

Occupational noise exposure is elevated for people working with machines such as foggers and ultra-low volume (ULV) aerosol devices [14,15,16] that have noise levels higher than 85 dBA, which is the Occupational Safety and Health Administration (OSHA) action level (AL) for an eight-hour time-weighted average (TWA) [17]. Hence, this could potentially lead to noise-induced hearing loss (NIHL) if the proper personal protective equipment (PPE) is not utilized [17]. It is mandated by OSHA that workers are not around noise levels >85 dBA more than eight hours per day without proper PPE [18,19]. In cases where noise levels are greater than 100 dBA, workers should only be allowed to be in that area for a total of two hours per day [18].

If proper PPE is not worn, insecticide exposure could lead to acute skin and/or respiratory exposure and/or chronic health problems [20]. However, proper PPE use protects against and reduces health risks, including acute and/or chronic effects [20]. Formulated insecticide products in the pyrethroid class are commonly utilized in mosquito control operations [3]. Ultra-low volume applications of pyrethrin and synthetic pyrethroid adulticides do not cause increased asthma symptoms, emergency department visits, or other acute illnesses, and biomonitoring studies have shown minimal systemic exposure [21,22]. A study on health effects of organophosphate adulticides used in mosquito control programs showed no evidence of increased metabolic toxicity, emergency visits, respiratory illness, or significant dermal absorption following malathion or naled applications according to EPA label instructions [23].

Sun exposure (i.e., UV) can occur while working outdoors during mosquito surveillance and other daytime mosquito control activities (e.g., setting and retrieving traps, conducting barrier treatments, larviciding, source reduction, and/or communicating with the public). Heat-related illnesses (e.g., heat stroke, heat exhaustion, heat cramps, heat rash, sunburn) can also occur in outdoor workers [24,25]. In warm regions, heat-related illnesses can be increased, depending on the workplace environment, specific work activities, and PPE worn [26,27]. When workers carry heavy equipment, they exert additional physical effort, thereby increasing the possibility of heat-related health effects such as dehydration and heat stress [28]. Furthermore, outdoor workers are at a greater risk of vector-borne diseases since they are exposed to potential vectors that may transmit pathogens (e.g., viruses, protozoans) to vertebrate hosts through blood feeding [25].

There is currently limited published information on the health and safety risks and mitigation plans of mosquito control personnel. There are more than 700 mosquito control programs at county, city, or state levels across the United States in addition to hundreds of private programs that conduct control activities in residential communities and commercial areas [29]. If proper safety measures are followed and routine up-to-date training is implemented, health and safety risks can be minimized and/or eliminated for these essential environmental/public health personnel. Here, health and safety training, workplace practices, as well as health and safety perceptions were surveyed for mosquito control personnel in NC and VA, and recommendations are discussed based on the findings.

## 2. Materials and Methods

A 28-question survey (Appendix A) was developed and classified as exempt by the East Carolina University Medical Center Institutional Review Board (UMCIRB# 23-001190). Once questions were designed and prior to dissemination, the questionnaire was administered to a local mosquito control program manager to help with validation. Questions were reassessed and then re-worded based on this feedback and the questionnaire was readministered to the mosquito control program manager a second time to check for consistency in answers before widespread administration to other programs. The exploratory baseline survey was administered through Research Electronic Data Capture (REDCap, version 16.0.30) [30,31]. Members (N = 213 people) of the NC Mosquito and Vector Control Association (NCMVCA; www.ncmvca.org, accessed on 4 June 2026) and VA Mosquito Control Association (VMCA; https://mosquito-va.org/, accessed on 4 June 2026) were surveyed electronically (i.e., email or QR code) between August 2023 and January 2024. These states are located in the mid-Atlantic eastern region of the United States. A USD 20 Amazon gift card was offered to respondents to encourage participation.

Statistical Package for Social Sciences (SPSS, version 28.0.1.1, IBM, Armonk, NY, USA) was used for statistical analyses. A descriptive analysis was carried out for data collected from the survey. Some questions used a Likert scale and these data were displayed using cross tabulation. Cross-tabulation tables were created to show the relationship between (1) *equipment noise* and *hearing*, (2) *slips*, *trips*, *falls* and *wearing a backpack sprayer*, and (3) *concern about insecticide health effects* and *attendance at safety training*. Due to the small sample size, regression analyses were not included.

## 3. Results

Sixty individual survey responses were received (28% response rate). Table 1 shows different job position titles held by respondents within mosquito control programs. The most common positions were mosquito control and treatment technician (n = 15, 25%) and environmental health specialist (n = 13, 22%) (Table 1).

Eight years (range two months to thirty-four years) was the average time respondents reported working in the mosquito control field. Most (95%) survey respondents worked with city or county mosquito control programs (MCP) and most (97%) reported their job was primarily focused on mosquito control. It was noted that 48% (n = 29) reported re-certification of their applicator license annually and 25% (n = 15) reported recertification every two to three years.

### 3.1. Occupational Hazards

Most workers (73%, n = 44) had not experienced a heat-related illness. However, 12% (n = 7) had experienced a heat-related illness and 8% (n = 5) reported being unsure. Most (83%; n = 50) strongly agreed or agreed about the importance of staying hydrated during the work day, while 8% (n = 5) strongly disagreed or disagreed. Respondents commented that vehicle air conditioning was important for staying cool during the work day.

Respondents were asked to classify their equipment noise as: (1) quiet (e.g., refrigerator, rain), (2) relatively loud (e.g., vacuum cleaner, traffic), or (3) very loud (e.g., motorcycle, gas lawn mower). Most (58%, n = 35) thought their equipment was relatively or very loud and 70% (n = 42) reported needing to raise the volume of their voice when talking to others during equipment use. Four respondents (7%) considered their equipment quiet. Some (18%; n = 11) had experienced ringing in their ears and/or muffled hearing after equipment use. Some (20%; n = 12) respondents commented that their hearing function may have been affected by loud work equipment. Table 2 shows an inconclusive relationship between reported equipment noise and hearing issues such as ringing and this could be complicated by the use of hearing protection.

Here, 37% (n = 22) of respondents described having slipped, tripped, or fallen at work. Some comments included: “fell in boot high mud,” “tip overs,” “trip a lot in woods”. Workers who used *backpack sprayers* were 16 times more likely to report a *slip, trip, or fall* compared to those who did not use a backpack sprayer (OR = 16.07, 95% CI [4.09–63.23]). However, the current survey did not specifically determine if the slip, trip, or fall occurred during backpack sprayer use and this should be investigated further in future work as the correlation/causality relationship was not explored here. Table 3 shows an inconclusive relationship between *slip*, *trip*, *fall* and wearing a *backpack sprayer*. There were 12 participants (20%) that reported having back pain after backpack sprayer usage.

More than half (55%; n = 33) of respondents expressed concern about the potential health effects of insecticide exposure. Despite concern, we noted that 39% of applicators did not wear a respirator during barrier treatments. The N95, KN95, and organic vapor aerosol types of respirators were the most utilized by respondents who wore respirators. Those that were concerned about insecticide health effects were likely to attend safety training (Table 4).

### 3.2. Mosquito Control Methods

In NC and VA, mosquito control personnel primarily work outdoors during May-September. Many respondents (67%, n = 40) used larvicides and conducted oviposition site source reduction (38%, n = 23), such as removing water-holding containers and improving drainage in ditches and other water-holding areas. One respondent indicated that adulticide usage occurred at their agency only after hurricanes when substantial flooding occurred. Another respondent reported that larvicides and/or adulticides were applied only upon reaching pre-determined threshold numbers established through surveillance (e.g., quantifying abundance of mosquito populations via adult trapping or larval dipping).

### 3.3. Mosquito Control Equipment Usage

Truck-mounted ULV machines (foggers) were the most common equipment (70%, n = 42) used for mosquito control, followed by backpack sprayers (42%, n = 25) and ATVs for wide area barrier treatments (25%, n = 15). Some indicated not conducting barrier (25%, n = 15) or ULV (22%, n = 13%) treatments in their mosquito control programs. Respondents indicated that a backpack sprayer weighed 11–32 kg; however, it was unclear whether that weight also included the formulated insecticide product. One person commented that their backpack sprayer weighed 31 kg, including the backpack [13 kg] and formulated insecticide product [18 kg]. Operators used backpack sprayers for approximately one hour per treatment with 13 treatments conducted per year per operator.

### 3.4. Precautions to Prevent Insecticide Exposure

Respondents reported following these precautions while working with insecticides: (1) following the label (80%, n = 48), (2) wearing non-absorbent gloves (73%, n = 44), (3) wearing long sleeved shirts (72%, n = 43), (4) not smoking or eating during preparation of and/or application (72%, n = 43) (5) washing exposed skin after application (68%, n = 41), (6) wearing non-absorbent shoes (55%, n = 33), and (7) respirator use (43%, n = 26). Employer-supplied PPE reported by respondents are shown in Table 5.

### 3.5. Worker Safety Training by Employers

Most (77%, n = 46) participated in employer training covering multiple topics including heat stress, safety (slips, trips, falls, cuts), ergonomics (lifting, posture, motion), insecticide handling, noise, UV radiation, and vibration. Respondents also reported receiving supplementary training on a variety of topics (e.g., driving safety, hazard communication, fire safety, first aid, active shooter response, Resource Conservation and Recovery Act, spill cleanup, private land access). Several training delivery methods were noted by preference: (77%, n = 46) in person, virtual live (58%, n = 35), written protocol (57%, n = 34), YouTube videos (55%, n = 33).

## 4. Discussion

To our knowledge, this pilot study is the first to assess occupational health and safety in mosquito control personnel in the United States. Mosquito control personnel are a highly trained and essential group of workers, defending public health against mosquito-borne and other vector-borne diseases, as well as helping control the occurrence and abundance of mosquito populations after flooding events to protect emergency workers and the public [4,32]. In NC and VA (eastern United States), the period of May to September represents the bulk of the mosquito season each year; however, this timing may vary in other areas, with an extended season in more southern regions [1,3]. In some mosquito control programs, temporary part-time or full-time seasonal workers are often hired during the busiest summer months to help with routine surveillance and other lab and field activities. The bulk of mosquito surveillance and control activities occur during the hottest times of year, putting workers at risk of heat-related illnesses; hence, workers should be aware of this and reminded to stay hydrated to prevent health effects [24,27,33]. Here, most survey respondents indicated that they stayed hydrated during the workday. Common potential hazards to outdoor workers include chemical exposure, heat stress, and injury [9,19,34]. These hazards are impacted by factors such as length and type or rigor of work activity, as well as heat acclimatization and physical condition of the person carrying out the activity [33]. The use of activity-specific PPE is recommended to reduce or prevent worker exposure to hazards when engineering and administrative controls are not feasible [19,33,34]. Mosquito control personnel in the current study reported using PPE (e.g., gloves, repellent, hearing protection, respirator) supplied by their employers and took appropriate safety precautions, such as following insecticide label instructions (e.g., handling, application rate, application method, clean up, disposal) to ensure safety.

The number and timing of continuing education hours required varied here, depending on the type of applicator license and state of the issued license. In NC, applicators must earn six hours of continuing education credits every five years to maintain their public health pesticide applicator license [7]. In VA, applicators must attend an approved course (no indicated time limit) that includes legal aspects and category-related information every two years to maintain each category of their pesticide applicator license [8]. Here, respondents indicated carrying out recertification activities every one to three years to fulfil the requirements of applicator license maintenance.

Surveillance-based targeted larvicide or adulticide applications using various types of handheld, backpack, ATV, and/or truck-mounted equipment may be necessary and are an identified component of IMM [35,36,37]. Here, most programs utilized truck-mounted ULV sprayers, while fewer than half of respondents reported utilizing backpack sprayers. Some programs reported focusing primarily on public education and outreach activities, rather than mosquito surveillance and/or control measures, while other programs reported a combination of these two approaches. Others have shown that carrying something heavy while performing a task can result in slips, trips, or falls [38]. The most reported issues related to carrying a backpack sprayer are fatigue and lower back pain [12]. Vibrations can occur from blower motors on backpack sprayers and this can contribute to back and other physical health problems [12]. Mosquito control operators should plan to take breaks periodically as a mitigation step to this potential health issue.

Here, the occurrence of slips, trips, and/or falls was higher in those using a backpack sprayer, compared to those who did not use this equipment. However, the correlation/causality relationship for these two variables was not explored in the current survey. This should be considered when interpreting results and further explored in future studies. Uneven terrain, fallen branches, muddy ground, and holes in the ground were noted by respondents as contributing to falls in their workday. In the United States, work-related slips, trips, and/or falls cost an estimated USD 9.19 billion annually in direct costs [38,39,40]. Slips, trips, and/or falls are preventable, and workers should be trained in situational awareness and to identify hazards at each different work site [40]. When walking in wooded areas on uneven ground, personnel should clear walkways of debris, wear sturdy non-slip footwear, walk slowly, and look ahead to scan the area for potential hazards [41]. This is especially important for mosquito control workers as their worksite is constantly changing as they conduct mosquito surveillance and control measures in different outdoor locations across their region of work. In some cases, unmanned aerial vehicles can assist in mosquito surveillance and control activities to reduce the need for workers to traverse uneven and potentially dangerous landscapes [42] and the extent of usage of these tools by programs should be evaluated in future studies.

Although some indicated working with loud equipment, respondents were generally not concerned about hearing loss. However, loss of hearing may result after many years of noise exposure; hence, workers using loud equipment should wear hearing protection to prevent potential hearing loss in the future [43]. Other workers have reported health concerns when mixing and/or applying insecticides [44,45]. Here, a relationship was identified between concern about insecticide exposure and the use of respirators, indicating that safety behavior may be influenced by one’s perception of health outcomes. This should be studied further in future studies with increased sample size. Formulated insecticide product label instructions should always be followed, and respirators may or may not be required, depending on the type of product. The label should be studied for each different product being used in mosquito control, with appropriate guidelines and training provided to personnel. Here, everyone working with insecticides reported wearing gloves, indicating that workers understand that PPE prevents dermal exposure. Conversely, a survey of mosquito control personnel in Pakistan found that many workers did not use proper PPE (e.g., mask, safety glasses, gloves, repellent), even though they were concerned about safety during insecticide applications (e.g., residual treatments and fogging) [46]. In some cases, this could be attributed to a lack of available PPE and/or a lack of sufficient training on safety precautions in that group of workers in Pakistan.

It is apparent that health and safety worker training increases awareness and can help increase workplace safety [20,41]. When employees are required to use PPE, such as when using insecticides or working with loud equipment, employers must implement a PPE employee training program [18,19,20]. It is unlikely that mosquito control workers are exposed to an eight-hour time-weighted average of noise that is ≥85 dBA (hearing conservation program required under these conditions) [19]; however, employee training on hearing protection and other PPE is recommended for those working with loud equipment such as backpack sprayers or ULV machines. In addition to employer-provided activity-specific training and in person or other educational conferences, there are many free online training options for mosquito control personnel including topics on IMM, risk communication, vector-borne diseases, policy development, and others that could be useful to employers and employees [4,47,48,49]. Here, respondents were trained by employers on aspects of health and safety and preferred the in-person training format, although virtual options were also used. It is essential to employ various training modalities, as individuals may exhibit different learning preferences.

## 5. Conclusions

This is considered a pilot/exploratory study given the small number of respondents (N = 60) from two states; hence, the findings may not be generalized to the entire country. Self-reported survey data should be interpreted with caution due to the possibility of recall bias. Workers concerned with safety may have been more prone to participate in the survey and this should be considered when interpreting results. Investigators attempted to minimize non-response bias by offering an incentive (gift card) to respondents, administering the survey by email and in person via QR code, and sending repeated reminders. Results pertain to self-reported outcomes and perceived exposure rather than measured exposure levels. The current study did not consider work experience, physical condition, ergonomic factors, worker fatigue, or other biomechanical risks that could lead to injury. This work could be expanded to include additional mosquito control workers and regions. It is possible that workers with chronic health issues (e.g., hearing impairment or back/spine problems) may leave this profession earlier due to the physical demands of some positions, which could have skewed the study’s results, particularly considering the average employment duration of eight years (although the range of employment duration was reported up to 34 years).

This baseline work can be considered a starting point for implementing and strengthening occupational safety and health awareness and preventive measures for mosquito control workers. Knowledge of health and safety hazards can help reduce workplace risk. Mosquito control personnel are essential workers that are paramount to public health, preventing vector-borne diseases, responding to natural disasters such as hurricanes, and conducting community awareness campaigns to mitigate risk. Here, potential hazard-creating situations were identified including working with loud equipment, insecticides, uneven terrain, and heat. Multiple types of PPE and training were reported that may help reduce worker risk. If further information is desired, employers could consider consulting with occupational health and safety professionals to assess risk and put preventative measures in place for mosquito control employees conducting various types of activities. Regular ergonomic breaks or mechanical aids should be considered in the workday planning process to relieve spinal strain, especially when working with heavy backpack sprayers (>30 kg). Additional research could include identifying work activities with potential risks (e.g., handling insecticides and other chemicals, working with loud equipment, carrying heavy equipment while walking), measuring and monitoring the risks associated with each activity (e.g., insecticide exposure, hearing effects, back pain or falls), implementing targeted training interventions for workers, and then evaluating subsequent improvements in worker health and safety.

## Figures and Tables

**Table 1:** Employment positions of mosquito control survey respondents.

Position	No. of Respondents	% of Respondents
Vector Control Personnel (Includes Mosquito Control and Treatment Technician)	15	25
Environmental Health Specialist (Includes REHS)	13	21.667
Environmental Health Supervisor/Program Manager	6	10
Vector Control Supervisor/Program Manager	5	8.333
Biologist	5	8.333
Operations Superintendent	3	5
Laboratory/Researcher	3	5
Sanitation Operations Supervisor	2	3.333
Entomologist	2	3.333
Environmental Health Director	1	1.667
Contractual Agricultural Inspector II	1	1.667
Public Works (City) Crew Leader	1	1.667
Pesticide Officer	1	1.667
Supervisor	1	1.667
No Answer	1	1.667
Total respondents	60	

**Table 2:** Relationship between equipment noise level and hearing.

		Hearing (Ringing in Ears)			
		Yes	No	Unsure	Total
Equipment Noise Level	Quiet	0	4	0	4
	Moderately Loud	2	10	0	12
	Very Loud	8	13	1	22
	Total	10	27	1	38

**Table 3:** Relationship between wearing a backpack sprayer and slips, trips, and falls.

		Slips, Trips, and Falls		
		Yes	No	Total
Wearing a Backpack Sprayer	Yes	10	5	15
	No	12	27	39
	Total	22	32	54

**Table 4:** Relationship between attendance at safety training and concern about insecticide health effects.

		Concern About Insecticide Health Effects		
		Yes	No	Total
Attendance at Safety Training	Yes	23	22	45
	No	7	1	8
	Total	30	23	53

**Table 5:** Personal protective equipment provided by employers.

Personal Protective Equipment	No. of Respondents	% of Respondents
Gloves	53	88
Repellent	50	83
Hearing Protection	42	70
Respirator	41	68
Eye Protection	16	27
Boots or Other Footwear	11	18
Long Sleeved Shirts or Pants	7	12
Face Shield	4	7
Apron	3	5
Mixing Sleeves	3	5
Permethrin-treated Clothing	3	5
Jacket	2	3

## Data Availability

Data available upon request.

## Abbreviations

The following abbreviations are used in this manuscript:

MCP	Mosquito Control Program
NIOSH	National Institute of Occupational Safety and Health
OSHA	Occupational Safety and Health Administration
IMM	Integrated Mosquito Management
UV	Ultraviolet
ULV	Ultra-low volume
NIHL	Noise induced hearing loss
PPE	Personal protective equipment
EPA	Environmental Protection Agency
NCMVCA	North Carolina Mosquito and Vector Control Association
VMCA	Virginia Mosquito Control Association

## Appendix A. Online Survey Distributed Through REDCAP System

Thank you for taking the time to participate in our North Carolina mosquito control program survey about occupational health and safety. The goal is to identify knowledge, attitudes, and practices regarding occupational tasks to identify potential areas for improvement, future study, and professional training. We plan to publish these results in a peer-reviewed journal. However, your participation will always remain anonymous, and your comments or answers will not be attributed to you individually in any form. You are not required to answer the questionnaire and will not be penalized in any way if you choose not to participate. To encourage participation, we would like to send you a $20 Amazon gift card. There is a space for open comments provided at the end of the survey. You may use this space to provide additional comments about any of the questions.

**Demographics**

(1) Please fill in the following information.
  City
  County
  Name of agency where you work
  Position title
  Description of job tasks and responsibilities
  Number of years in current position
  Email address (where gift card will be sent)
(2) About how long has your program been conducting mosquito surveillance and/or control?
  - >20 years
  - 10–20 years
  - 5–9 years
  - 2–4 years
  - 1 year or less
  - Unsure
  - My program does not conduct mosquito surveillance or control, but we do community education about mosquitoes.
    Additional comments_____________________________
(3) How often are you re-certified as a pesticide applicator?
  - Every 6 months
  - Every year
  - Every 2–3 years
  - Other (describe)______________

**Equipment used**

(4) When do you use mosquito control (for what purpose and how often)?
  (select all that apply and add comments, as needed)
  - Use larvicides numerous times during the year to control immature mosquitoes
  - Use adulticides numerous times during the year to control adult mosquitoes
  - Conduct source reduction (e.g., removing containers, improving drainage)
  - My program does not conduct mosquito control
    Additional comments_____________________________
(5) What type of equipment do you use to apply pesticides used in mosquito control?
  (select all that apply and add comments, as needed)
  - Backpack sprayer for back yard barrier and other treatments
  - Sprayer mounted on ATV for barrier or area wide treatments
  - Truck-mounted ultra-low volume (ULV) machine for area wide treatments
  - My program does not conduct barrier treatments
  - My program does not conduct ULV treatments
    Additional comments on equipment used to apply pesticides or other equipment used at work _____________________________
(6) If you use a backpack sprayer, estimate the size and weight of this equipment and duration of use at each application and/or day.
  - Size ________________________
  - Weight ______________________
  - Average duration (minutes) of use at each application _____________________
  - Number of applications per month during mosquito season________________
(7) Is your equipment too heavy?
  - Yes
  - No
    Additional comments_______________________________________________
(8) Rate the perceived noise level of your equipment.
  - Quiet (i.e., refrigerator hum, light rain)
  - Moderately loud (i.e., vacuum, moderate traffic, noisy restaurant)
  - Very loud (i.e., motorcycle, gas powered lawn mower)
    Additional comments_____________________________

**Personal Protective Equipment Usage**

(9) Please list the brand name and/or types of pesticides you use in barrier and/or ULV treatments (if any).
  ____________________________________________________________
(10) Do you use a respirator during back yard barrier treatments? If yes, what type? If not, why not?
  - No
  - Yes—organic vapor/aerosol respirator
  - Yes—N95/ KN95
  - Yes—surgical mask
  - Yes—other (specify: __________________)
  - I do not conduct barrier treatments.
(11) Do you use hearing protection when using the backpack sprayer for barrier treatments? If yes, what type? How often?
  - No
  - Yes—ear plugs
  - Yes—ear muffs
  - Yes—other (specify: _____________________)
  - I do not conduct barrier treatments.
(12) Do you use gloves when pouring pesticide products into equipment (e.g., backpack sprayer or ULV machine) for use? If yes, what type of gloves? If not, why not?
  - No; specify why not______________________
  - Yes; specify type of gloves: __________________________
  - I do not use pesticides.
(13) What precautions do you take to prevent pesticide exposure? (please check all that apply)
  - Wear long sleeves and pants
  - Wear non-absorbent shoes during application
  - Wear non-absorbent gloves during application
  - Use a respirator during pesticide handling and application
  - Follow instructions on pesticide label
  - Wash exposed skin after application
  - Not smoking or eating during pesticide handling
  - Other _______________________
    Additional comments
(14) My employer provides (select all that apply):
  - Hearing protection
  - Respirator
  - Gloves
  - Repellent
  - Other personal protective equipment (please list)
    Additional comments_____________________________
(15) I make sure to stay hydrated by drinking water throughout the day when I am working outdoors.
  - Strongly Disagree
  - Disagree
  - Neither Agree nor Disagree
  - Agree
  - Strongly Agree

**Health Effects and Concerns**

(16) Have you experienced any heat-related illness or issue (e.g., heat stroke, heat exhaustion, fainting, heat cramps) during your workday?
  - Yes
  - No
  - Unsure
    Additional comments_____________________________
(17) Do you experience ringing in your ears or muffling of sound after equipment use?
  - Yes
  - No
  - Unsure
    Additional comments_____________________________
(18) Do you think your hearing is affected by your equipment use?
  - Yes
  - No
  - Unsure
    Additional comments_____________________________
(19) Do you have to raise your voice when talking to others while using the equipment?
  - Yes
  - No
(20) Have you experienced slips, trips or falls during work activities?
  - Yes; Please describe_______________________________
  - No
(21) Have you experienced back pain after wearing a backpack sprayer?
  - Yes
  - No
    Additional comments__________________________
(22) During the hottest time of the year, in which months do you work outdoors? Select all that apply
  - May
  - June
  - July
  - August
  - September
(23) Are you concerned about the health effects of pesticides?
  - Yes
  - No
    Additional comments_____________________________

**Safety Training**

(24) Have you attended a health and safety training conducted by your employer?
  - Yes
  - No
  - Unsure
(25) (If yes in item 24) What topics are covered in the health and safety training that you attended? (Select all that apply.)
  - Pesticide
  - Noise
  - Solar UV radiation
  - Vibration
  - Heat stress
  - Ergonomic issues (e.g., heavy lifting, awkward posture, repetitive motion)
  - Safety issue and injuries (e.g., falls, slips and trips, cuts)
  - Other___________________________
(26) (If yes in item 24) How often do you attend your employer’s health and safety training?
  - More than once a year (e.g., every 6 months)
  - Once a year
  - Once every 2 years
  - Other; specify _____________________________
(27) What kind of training would help you (and/or others) maximize health and safety for employees conducting mosquito surveillance and/or control?
  (select all that apply and add additional comments, as needed)
  - YouTube training videos
  - Hands-on face-to-face training
  - Live training in a virtual format
  - Manuals with detailed “step-by-step” health and safety instructions
    Additional comments____________________________
(28) Is there anything else you would like to add to this conversation about occupational health and safety? Please let us know if there is any topic about occupational health and safety that has not been addressed.
  _________________________________________________
